# Percutaneous Closure of PFO in Patients with Reduced Oxygen Saturation at Rest and during Exercise: Short- and Long-Term Results

**DOI:** 10.1155/2020/9813038

**Published:** 2020-03-20

**Authors:** Céline De Cuyper, Tristan Pauwels, Eric Derom, Michel De Pauw, Daniël De Wolf, Paul Vermeersch, An Van Berendoncks, Bernard Paelinck, Gaëlle Vermeersch

**Affiliations:** ^1^Department of Respiratory Medicine, Ghent University Hospital, Ghent, Belgium; ^2^Department of Cardiology, Ghent University Hospital, Ghent, Belgium; ^3^Department of Cardiology, Antwerp Cardiovascular Center, ZNA Middelheim, Antwerp, Belgium; ^4^Department of Cardiology, Antwerp University Hospital, Edegem, Belgium

## Abstract

**Background:**

A patent foramen ovale (PFO) is a rare cause of hypoxemia and clinical symptoms of dyspnea. Due to a right-to-left shunt, desaturated blood enters the systemic circulation in a subset of patients resulting in dyspnea and a subsequent reduction in quality of life (QoL). Percutaneous closure of PFO is the treatment of choice.

**Objectives:**

This retrospective multicentre study evaluates short- and long-term results of percutaneous closure of PFO in patients with dyspnea and/or reduced oxygen saturation.

**Methods:**

Patients with respiratory symptoms were selected from databases containing all patients percutaneously closed between January 2000 and September 2018. Improvement in dyspnea, oxygenation, and QoL was investigated using pre- and postprocedural lung function parameters and two postprocedural questionnaires (SF-36 and PFSDQ-M).

**Results:**

The average follow-up period was 36 [12–43] months, ranging from 0 months to 14 years. Percutaneous closure was successful in 15 of the 16 patients. All patients reported subjective improvement in dyspnea immediately after device deployment, consistent with their improvement in oxygen saturation (from 90 ± 6% to 94 [92–97%] on room air and in upright position) (*p* < 0.05). Both questionnaires also indicated an improvement of dyspnea and QoL after closure. The two early and two late deaths were unrelated to the procedure.

**Conclusion:**

PFO-related dyspnea and/or hypoxemia can be treated successfully with a percutaneous intervention with long-lasting benefits on oxygen saturation, dyspnea, and QoL.

## 1. Introduction

A foramen ovale is a flap-like opening between the right and left atrium of the heart during fetal life. It normally closes during infancy but remains patent (patent foramen ovale or PFO) in approximately 25% of humans. A minority of subjects with a PFO may develop clinical symptoms [1–4] such as a paradoxical embolus and is suspected to be even related to migraine and sleep apnea. More rarely, it may cause profound hypoxia and symptoms of dyspnea. Symptoms can occur late in life and may be precipitated by a cardiac or extracardiac event, such as a pneumonectomy with a shift of the mediastinum, aortic root aneurysm or elongation, kyphosis, and unilateral paralysis of the diaphragm [5]. These mechanical distortions may change the position of the atrial septum relative to the inferior vena cava, thereby significantly increasing the degree of shunting [6]. In symptomatic patients with PFO, percutaneous closure is the treatment of choice [3, 5, 7].

The aim of the current retrospective study was to evaluate patients suffering from PFO-related dyspnea the short- and long-term effects of percutaneous closure on dyspnea, physiological outcome, and quality of life.

## 2. Materials and Methods

### 2.1. Study Design and Patient Data

A multicentre study was performed in three Belgian hospitals: Ghent University Hospital (UZGent), University Hospital of Antwerp (UZA), and ZNA Middelheim, Antwerp (ZNA). Listings from the catheterization laboratories and the billing section of the hospital's pharmacist were used to identify potential cases. These were subsequently cross-referenced to exclude doubles and completed with cases that some cardiologists remembered. To be included in the study, the indication for PFO closure had to be dyspnea and/or hypoxemia in all the patients in order to include as many patients as possible. Patients with other indications for closure, for example, paradoxical embolus, decompression sickness in divers, or migraine, were excluded. Patient characteristics, such as age, gender, cardiovascular risk factors, and duration of complaints, were obtained by consulting the local electronic patient database or the paper patient files, classified and stored in a database to assess the pre- and postoperative clinical status.

### 2.2. Measurement of QoL

Data on QoL were obtained prospectively by sending the patients the validated translations in Dutch of two questionnaires by email: the Medical Outcomes Study 36-Item Short-Form Health Survey (SF-36) and the Pulmonary Functional Status and Dyspnea Questionnaire, Modified version (PFSDQ-M).

The SF-36 is a set of generic, coherent, and easily administered quality-of-life measures, consisting of eight domains: physical functioning, role limitations due to physical problems, bodily pain, general health, vitality, social functioning, and role limitations due to emotional problems and mental health. For each domain, the score ranges from 0 (worst) to 100 points (best). The results can be compared with the averages in the Medical Outcomes Study [8].

The PFSDQ-M was developed to quantify the experienced change in performing ADL compared with the period before disease onset and symptoms of dyspnea and fatigue related to ADL. The questionnaire analyzes ten common activities, for example, putting on a shirt and climbing the stairs. Scores range from 0 (no dyspnea) to 10 points (severe dyspnea). PFSDQ-M has been translated into eight languages and is used internationally to evaluate dyspnea [9].

The design of the study was approved by the Ethics Committee of UZGent, UZA, and ZNA on the following dates, respectively: August 4, 2017; March 26, 2018; and May 9, 2018.

### 2.3. Data Processing and Confidentiality

Data collection of the three hospitals was performed in the same way. First, the informed consent was sent to the patients. After their approval, the retrospective data were retrieved from the electronic patient database and the SF-36 and PFSDQ-M questionnaires were sent. Those who did not answer within one month received a reminder by email or by phone.

All patients signed an informed consent to participate and collected data were kept confidential. Refusing to participate without any justification had no impact on future care. Patients were informed that they could withdraw from the study at any stage without victimization or denial of treatment.

### 2.4. Statistical Analysis

IBM SPSS Statistics 24 (Statistical Package for the Social Sciences; IBM Corporation, Armonk NY) was used to process the parameters and questionnaires mentioned above, assess their distribution, calculate the averages, standard deviations, and quartiles, run the Wilcoxon tests, and create the charts.

The Shapiro–Wilk test, histograms, Q-Q plots, and boxplots were used to assess normality. Normally distributed data were expressed as mean (±SD) and nonnormally distributed data as mean and quartiles [Q_1_–Q_3_]. The Wilcoxon signed-rank test was used to analyze the difference between pre- and postprocedural parameters.

Since no questionnaires before closure have been taken, scores after PFO closure were compared with SF-36 scores of a Dutch standard population (by Kruijshaar et al. [10]) and with scores of a patient group with advanced COPD (by Janssen et al. [11]).

## 3. Results

### 3.1. Selection of Patients

Between January 2000 and September 2018, 1.287 patients underwent percutaneous PFO closure at UZGent, UZA, and ZNA.

**Figure 1:**
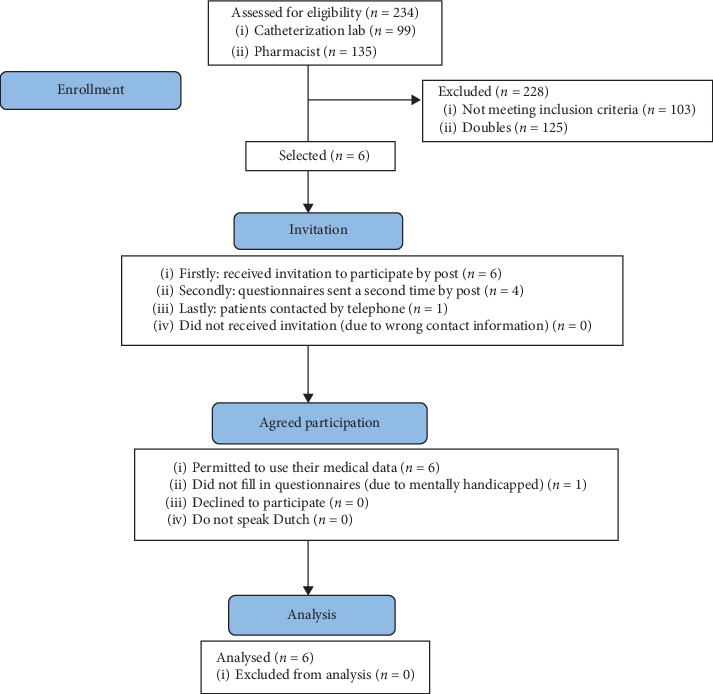
Consort diagram UZGent.

Identification of patients in UZGent was based on two lists: one from the catheterization lab (99 patients) and one from the pharmacist (135 patients). Doubles were excluded, 109 medical files remained, and only those patients in whom dyspnea and/or hypoxemia was the indication for PFO closure were retained. This ultimately led to the inclusion of six patients (Figure 1). Five of these returned their questionnaires. The mental disability (not related to PFO closure) of the sixth patient prevented her to fill in the questionnaires.

Cardiologists in charge of the catheterization laboratory at UZA provided data required for the present study. Two out of 387 patients underwent percutaneous PFO closure because of severe dyspnea. One patient had undergone the intervention before the digitalization of patient records. A case report with few demographic and functional data was the only source related to this patient and questionnaires were not sent due to missing contact details. As the other patient passed away, QOL was not obtained either.

The dataset of ZNA contained 791 PFO closures since January 1, 2000, from which eight patients were included in this study, of whom three had already passed away. Of the remaining five patients, only three filled in the questionnaires, whereas the treating physicians of the remaining two patients claimed that they were still in very good physical condition and did not exhibit any dyspnea. The lack of digitalization of patient records caused the data from the three deceased patients to not be as extensive as most of the digitalized files. For one of these, some data were retrieved from a case report (personal communication) [12].

Eventually, the database contained data of 16 patients, of whom 12 were still alive and 8 filled in the two questionnaires. The devices used to close the PFO are listed in Table 1.

### 3.2. Patients' Baseline Characteristics and Follow-Up

Baseline characteristics of the 16 patients, expressed either as mean ± SD or mean [quartile 1–quartile 3], are presented in Table 2. Mean age [Q1–Q3] at time of closure was 59 [50–75] years and 50% of patients were female. Mean (±SD) New York Heart Association Functional Classification (NYHA) was 3.0 ± 0.8.

The most relevant associated medical conditions are summarized in Table 3. Pneumonectomy was the most common surgical procedure (*n* = 2). The average duration [Q1–Q3] of dyspnea before percutaneous intervention was 6 [1–8] months, ranging from a few days to 24 months.

The average follow-up period was 36 [12–42] months, with a range of 0–181 months and 6 patients being lost to follow-up. Two early deaths were caused by acute respiratory failure on top of a preexisting chronic respiratory failure of pulmonary etiology and bronchial cancer. Among the two late deaths, one was attributed to a carcinoid tumor and the other one being of unknown origin.

### 3.3. Postprocedural Physiological Outcomes

14 of 15 patients with successful closure reported complete resolution of their dyspnea complaints after closure. One patient had an initial improvement, but three months after closure she experienced shortness of breath again, which was ultimately not considered as a consequence of her PFO. Moreover, oxygen saturation improved statistically significantly (*p*=0.014) immediately after device deployment (preprocedure: 90.2 ± 6%; postprocedure: 94.0% [92%–97%] on room air). Percutaneous closure of a PFO had, however, no substantial impact on the other outcomes such as PaO_2_, PaCO_2_ or pulmonary function parameters, although a trend toward statistical significance (*p*=0.08) was seen for PaO_2_ (Table 4).

In one of the 16 patients a small residual clinically irrelevant left-to-right shunt across the occluder device could be identified. That patient was readmitted for a redo procedure, which was not successful because of technical issues. Repeat interventions were not required in the remaining 15 patients and major complications were not recorded. One patient experienced pain at the femoral access site and had a transient reduction in hemoglobin shortly after the procedure, which was uneventful. Long-term adverse events suggesting device malfunction were not reported.

### 3.4. Quality of Life

**Figure 2:**
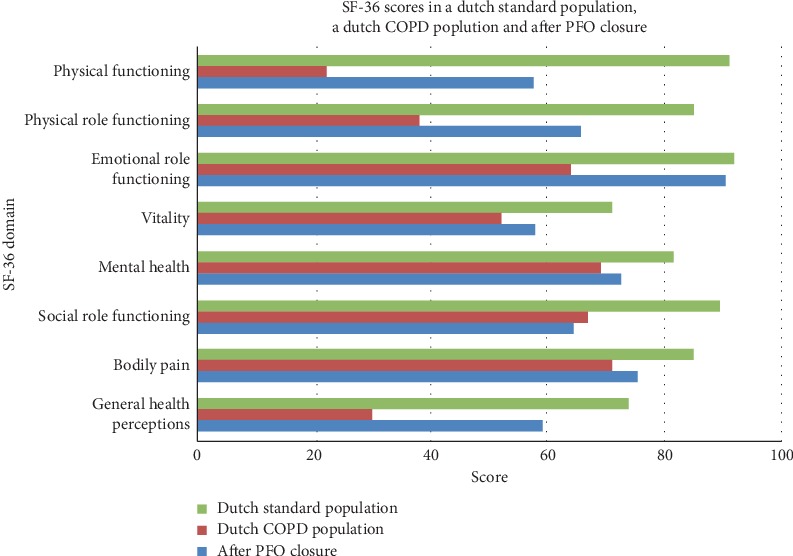
Statistical analysis of SF-36 questionnaire in 8 patients after PFO closure. A slight impairment in quality of life (QoL) is seen in comparison to a standard population. Scores after PFO closure are superior to the scores from patients with advanced COPD.

Mean scores of the patients after PFO closure for the different domains of the SF-36 are shown in Figure 2. Mean score was 57.5/100 for physical functioning, 65.6/100 for role limitations due to physical health, and 59.3/100 for general health. Overall, all scores after closure exceeded the threshold of 50 points, which corresponds with the general population norm according to Kruijshaar et al., thus indicating a favorable change in QoL [10]. Health status after PFO closure was lower compared to the standard population, but better than in the COPD population. The lower score in QoL compared to that of the standard population could in part be attributed to 2 out of the 8 patients with severe osteoporosis and dyspnea due to a poor physical condition. These comorbidities negatively affected the averages, since the remaining 6 patients reported scores that approximated the averages of the standard population. QoL as assessed with SF-36 of patients after PFO closure invariably exceeded that seen in COPD patients [11].

The PFSDQ-M questionnaire, used to evaluate the change in dyspnea when performing activities of daily life, increased by 2.05 ± 2.56 points after closure, which corresponded to a slight improvement. When asked about the current degree of dyspnea and fatigue experienced during most days of the year, the average scores for dyspnea and fatigue were 1.90 ± 1.97 and 2.71 ± 3.48, respectively, corresponding to a mild degree of fatigue and dyspnea in daily life.

## 4. Discussion

This study of the impact of percutaneous PFO closure in 15 of the 16 patients not only demonstrates that the intervention leads to an immediate improvement in oxygen saturation and reduction of dyspnea after device deployment but is the first to evaluate the long-term effect of percutaneous PFO closure on QoL.

Over the last two decades, only five case series regarding percutaneous PFO closure in patients with dyspnea have been published (Table 5). Only two of these contained substantially more patients than the present series [5, 13–16]. These studies are not completely comparable to the current one, as some series contain only patients with platypnea-orthopnea syndrome (POS), while others included only patients with hypoxemia [5, 13–16]. Our study included all patients with PFO-related dyspnea and/or hypoxemia. Moreover, the present study—in contrast with the previous series—has a mean duration of follow-up of 36 months (range: 0 months–14 years) and ranged between 11 and 26 months in the previous ones. This longer follow-up period of up to 14 years not only allowed concluding the long-term safety of PFO closure in a context of dyspnea and/or hypoxemia, but also convincingly demonstrates that the beneficial effects of PFO closure in terms of QoL do not wean away after several years.

Surprisingly, patient-reported outcomes have never been investigated in this population. Our findings support that PFO closure is not only an effective treatment of respiratory symptoms but also yields long-lasting beneficial effects. More specifically, QoL assessed with the SF-36 indicated that most patients after PFO closure experienced a QoL which exceeds that of COPD patients and almost equaled that of a standard population.

The improvement in SaO_2_ seen in the current study was smaller than what has been reported in previous studies [5, 13–16]. In these studies, however, preprocedural mean SaO_2_ was lower, and, hence, there was more room for improvement. The improvements in SaO_2_ observed in the current study did not translate into significant changes in PaO_2_. This could be attributed to the small sample size or a few confounding factors such as the scatter of the preprocedural PaO_2_ and the comorbidities mentioned in the next paragraph. Nevertheless, a trend toward a statistical significance (*p*=0.08) was observed.

PFO can be the cause of dyspnea in patients having a normal SaO_2_. In a part of the patients, the respiratory center will react on hypoxemia, causing hyperventilation and dyspnea. Dyspnea increases the tidal volume and thus normalizes the arterial saturation. As the reaction of the respiratory center on hypoxemia can differ, other patients will not react on hypoxemia and not become dyspneic [17].

A large number of patients included in the present study suffered from severe cardiopulmonary comorbidities, a finding also reported in other studies. Interestingly, pneumonectomy, ascending aorta aneurysm (or dilation), and right hemidiaphragm elevation were also reported in the four other studies, but not in that of Ilkhanoff [5, 13–16]. However, in the latter study, almost all patients suffered from chronic pulmonary disease and congestive heart failure.

The technique of percutaneous PFO closure has been reported to be safe, and the present data confirm this finding [5, 13–16]. Indeed, no major procedure-related complications were observed and the overall mortality was not related to the intervention. It is reasonable to attribute our success rate and safety data of the PFO closing technique to the large experience of the catheterization laboratories of the three institutions involved, as these have performed more than 1200 similar procedures over the last 18 years.

### 4.1. Limitations

Although the long duration of follow-up definitely represents a strength, this study has several limitations. Firstly, this is a cross-sectional, retrospective study. Moreover, PFO-related dyspnea can be considered as an “orphan disorder,” and it is very unlikely that a randomized clinical trial will ever be conducted to prove the efficacy of PFO closure in patients with respiratory symptoms. A second limitation of our study is that some parameters were lacking due to the incompleteness of several patient records or death. As a consequence, only eight patients could fill in the questionnaires. All questionnaires were sent to the patients at the same point of time, causing vast differences in time interval between PFO closure and assessment of QoL. Moreover, confounding factors, such as the comorbidities described in Table 3, might have affected QoL measurement, particularly since a PFO-specific questionnaire has not been developed so far, and the results of the SF-36 and PFSDQ-M questionnaires are sensitive to pick up reductions in QoL due to a variety of symptoms caused by other diseases. For example, the eventual score of the SF-36 questionnaire in a patient with severe osteoporosis was definitely more affected by that aforementioned disorder than by a PFO-related dyspnea. Finally, missing parameters in the study population, a typical feature of retrospective studies, render the interpretation of some of the physiological data somewhat problematic. Since it is very unlikely that randomized controlled studies will ever be conducted in patients undergoing a PFO closure because of dyspnea and/or hypoxemia, cardiologists in charge of such patient should be invited to design a national or even international multicentre cohort study in which relevant data on procedural outcome in patients with a closed PFO would be collected prospectively.

The limited sample size did not allow for a statistical comparison of the QoL between the patients with low saturation and those with normal saturation before the intervention. The aim was to include all patients with PFO-related dyspnea and low oxygen saturation. The mentioned comparison is beyond the scope of the current study.

## 5. Conclusion

In conclusion, patients with PFO and reduced oxygen saturation at rest and during exercise benefit from percutaneous PFO closure. The PFO closure not only resulted in immediate increase in systemic arterial saturation and immediate improvement of the patients' dyspnea status. The procedure is safe and leads to a long-lasting improvement of dyspnea and QoL during long time follow-up. Overall, percutaneous PFO closure can be recommended to patients suffering from PFO-related dyspnea and/or hypoxemia at rest or during exercise.

## Figures and Tables

**Table 1:** Used devices.

Occlutech Figulla® flex II occluder	6 (37.50)
AMPLATZER™ septal occluder	3 (18.75)
Nit-occlud®	2 (12.50)
Hyperion™ PFO occluder	1 (6.25)
STARFlex occluder	1 (6.25)

Values are *n* (%).

**Table 2:** Baseline characteristics.

Age, years	59 [50–75]
Male/female	8/8
Alive	*n* = 10
Unknown	*n* = 1
BMI, kg/m^2^ [10]	25.2 ± 3.70
NYHA functional class [7]	3.0 ± 0.8
NYHA functional class (I/II/III/IV)	0/2/3/2
Duration dyspnea, months [9]	6 [1–8]
Follow-up, months	36 [12–42]
Spontaneous shunt [12]	*n* = 6
Cardiovascular risk factors	
Hypertension [8]	*n* = 3
Tobacco use, pack-years	10 [0–15]

Normally distributed values are mean ± SD; nonnormally distributed values are mean [Q_1_–Q_3_]. Numbers between brackets indicate number of patients from whom data that were obtained. BMI = body mass index; NYHA = New York Heart Association.

**Table 3:** Most relevant associated conditions.

Congenital malformations	7
Pectus excavatum	1
Pectus carinatum	1
Kyphoscoliosis	1
Diaphragmatic hernia	2
Pulmonary hypoplasia	1
Dextroversion	1
Pulmonary pathology	15
Obstructive diseases	6
COPD	1
Asthma	2
Air trapping and hyperinflation	1
Emphysema	1
Alpha-1 antitrypsin deficiency	1
Restrictive lung disease	9
Fibrothorax	1
Pneumonia	2
Pneumonectomy	2
Interstitial lung disease	1
Unspecified restrictive disease	1
Elevated right hemidiaphragm	2
Cardiovascular pathology	10
Pericarditis	1
Endocarditis lenta	1
Unfolded aorta	1
Dilated ascending aorta	1
DVT	1
CVA	1
Atrial fibrillation	1
Coronary artery bypass grafting	1
Acute myocardial infarct	1
Edema lower limbs	1
Oncologic pathology	4
Lung carcinoma	2
Benign tumor breast	1
Carcinoid carcinoma	1
Platypnea-orthodeoxia syndrome	2
Reflux esophagitis/Barrett's esophagus	3
Epilepsy	2

Values are *n*. COPD = chronic obstructive pulmonary disease; DVT = deep vein thrombosis; CVA = cerebrovascular accident.

**Table 4:** Pre- and postprocedural parameters.

	Before PFO closure	After PFO closure	*p* value
SaO_2_ standing (%)	90.2 ± 6.3	94.0 [92.0–97.0]	*0.014*
Unknown	*n* = 3	*n* = 5	
PaO_2_ (mmHg)	64.9 ± 14.4	77.8 ± 16.4	0.080
Unknown	*n* = 6	*n* = 6	
PaCO_2_ (mmHg)	33.7 [28.5–38.2]	36.8 ± 6.3	0.686
Unknown	*n* = 6	*n* = 11	
SaO_2_ standing after 6MWT (%)	82.8 [77.0–87.8]	92.7 ± 4.0	0.109
Unknown	*n* = 12	*n* = 13	
FEV1 (% of predicted)	92.0 ± 40.9	95.6 ± 55.6	0.271
Unknown	*n* = 7	*n* = 9	
FVC (% of predicted)	101.8 [75.8–127.2]	104.4 ± 44.6	0.237
Unknown	*n* = 7	*n* = 9	
Tiffeneau-Pinelli index (FEV1/FVC) (%)	77.6 ± 21.9	81.7 ± 29.5	0.866
Unknown	*n* = 6	*n* = 9	
PEF (% of predicted)	100.9 ± 30.7	97.7 ± 32.5	0.173
Unknown	*n* = 8	*n* = 10	
DLCO (% of predicted)	67.0 ± 14.4	64.6 ± 23.1	0.893
Unknown	*n* = 8	*n* = 10	

Normally distributed values are mean ± SD; nonnormally distributed values are mean [Q_1_–Q_3_]. SaO_2_ = oxygen saturation; PaO_2_ = partial pressure of oxygen; PaCO_2_ = partial pressure of carbon dioxide; 6MWT = six-minute walk test; FEV1 = forced expiratory volume in 1 second; FVC = forced vital capacity; PEF = peak expiratory flow; DLCO = diffusing capacity for carbon monoxide.

**Table 5:** Published series of PFO closure because of dyspnea or desaturation.

Author	Year	Number of patients	Mean age (years)	Closure rate	Absolute increase in SaO2	Major in-hospital complications	Mean follow-up period	Follow-up results
Guérin [13]	2005	78	67	97%	10%	2 unrelated deaths	16 m	7 late deaths (unrelated to procedure)
Shah [14]	2016	52	66	100%	14%	2 unrelated deaths, 1 AF, 1 VF	26 m	2 late AF
Mojadidi [5]	2015	17	63	94%	16%	-	11 m	64.8% improvement
Current study	2018	16	59	94%	4%	None	36 m	2 early and 2 late deaths (unrelated)
Ilkhanoff [15]	2005	10	63	100%	9%	1 TIA	—	—
Zavalloni [16]	2013	6	63	100% after redo	17%	1 unrelated death	3 m	1 TIA, 3 repeat interventions

—, missing; SaO_2,_ oxygen saturation; AF, atrial fibrillation; VF, ventricular fibrillation; TIA, transient ischemic attack.

## Data Availability

The data used to support the findings of this study are included within the article. The individual data on QoL, lung function parameters, and patient characteristics used to support the findings of this study are restricted by the Ethics Committee of UZGent, UZA, and ZNA in order to protect patient privacy. Data are available from Professor Dr. Eric Derom, eric.derom@uzgent.be, for researchers who meet the criteria for access to confidential data.

## Abbreviations

6MWT: Six-minute walk test
BMI: Body mass index
COPD: Chronic obstructive pulmonary disease
CVA: Cerebrovascular accident
DLCO: Diffusing capacity of the lung for carbon monoxide
DVT: Deep vein thrombosis
FEV1: Forced expiratory volume in one second
FVC: Forced vital capacity
NYHA: New York Heart Association Functional Classification
PaCO_2_: Arterial partial pressure of carbon dioxide
PaO_2_: Arterial partial pressure of oxygen
PEF: Peak expiratory flow
PFO: Patent foramen ovale
PFSDQ-M: Pulmonary functional status and dyspnea questionnaire, modified version
POS: Platypnea-orthodeoxia syndrome
QoL: Quality of life
SaO_2_: Oxygen saturation
SD: Standard deviation
SF-36: Medical Outcomes Study 36-Item Short-Form Health Survey
SPSS: Statistical Package for the Social Sciences.
